# Unequal goodbyes: disparities in end-of-life care before and after the outbreak of the COVID-19 pandemic across 28 countries

**DOI:** 10.1186/s12904-026-02220-y

**Published:** 2026-07-07

**Authors:** Paola Sillitti, Clément Meier, Ralf J. Jox, Jürgen Maurer

**Affiliations:** 1 Faculty of Biology and Medicine (FBM) & Faculty of Business and Economics (HEC), University of Lausanne, Swiss Centre of Expertise in the Social Sciences (FORS), Swiss Centre of Expertise in Life Course Research (LIVES), and Lausanne University Hospital (CHUV), Internef – Dorigny, Lausanne, 1015 Switzerland; 2 Palliative and Supportive Care Service, Chair in Geriatric Palliative Care, and Institute of Humanities in Medicine, Lausanne University Hospital and University of Lausanne, IHM - Av. de Provence 82, Lausanne, 1007 Switzerland; 3 Faculty of Business and Economics (HEC), University of Lausanne, and Swiss Centre of Expertise in Life Course Research (LIVES), Internef – Dorigny, Lausanne, 1015 Switzerland

**Keywords:** End of life, COVID-19, Pandemic, Healthcare, Older adults, Mortality, Disparities

## Abstract

**Background:**

The COVID-19 pandemic significantly disrupted health systems, impacting how individuals received end-of-life (EOL) care and amplifying pre-existing differences in healthcare use across sociodemographic groups. While research has examined the burden placed on healthcare professionals, less attention has been paid to the experience of care recipients in their final year of life, particularly across different socioeconomic groups. This study explores the sociodemographic factors associated with the care that people received in the last year of life, comparing periods before and after the outbreak of the pandemic across Europe.

**Methods:**

The analysis uses data from 5,029 deceased individuals aged over 50, who died between 2018 and 2022. Data include 28 European countries and are drawn from waves 7–9 of the Survey of Health, Ageing and Retirement in Europe. Information was collected via proxy interviews post-mortem and includes details on health, care received in the final year of life, and sociodemographic characteristics. The main outcomes were six binary variables indicating the utilization of care in the hospital, hospice, nursing home, home care, care from a general practitioner, and from a specialist physician. Using six binary probit regression models, we estimated the average marginal effects of dying after the onset of the pandemic (March 2020), with interaction terms for cause of death, education level, and financial difficulty.

**Results:**

EOL care use declined following the pandemic’s onset, with particularly steep reductions in home-based care and specialist services. These changes varied across population groups. Dying from cardiovascular or infectious causes was associated with significant declines in hospital, home, and specialist care use. Socioeconomic disparities widened: low education levels were associated with decreases across all care types except nursing homes, while reporting financial hardships was associated with marked reductions particularly in home care and specialist care use. Individuals with higher education or financial security saw smaller or no significant changes in care use.

**Conclusions:**

Patterns observed during the pandemic suggest that pre-existing differences in EOL care across Europe might have widened, and lower education and financial resources were associated with lower EOL care use. These results underscore the urgent need for policies that build resilient, equitable EOL care systems capable of protecting disadvantaged populations in times of crisis.

**Supplementary Information:**

The online version contains supplementary material available at https://doi.org/10.1186/s12904-026-02220-y.

## Background

The COVID-19 pandemic significantly disrupted health systems, which were often overwhelmed and insufficiently prepared to respond to the crisis. Growing care needs and lower availability of healthcare staff, as well as restrictions on visiting rights in institutional settings and growing use of digital health heavily impacted the way people received care and their experiences at the end of life [1–4]. To analyse how end-of-life care was disrupted during the pandemic, evidence is needed on how both the care providers and care recipients were affected by the health crisis.

Existing literature has extensively explored the impact of the pandemic on the physical and mental health status of health professionals, as well as the professional challenges they faced during this time, including the significant increase in workload and responsibilities, and a growing emotional toll linked to higher exposure to death [5–10].

To our knowledge, a smaller body of literature has explored how the pandemic disrupted the experience of care recipients, particularly for those at the end of their life, and how it impacted existing disparities in healthcare use at the end of life. While socioeconomic gradients of health and healthcare existed well before the COVID-19 pandemic, the latter represented an opportunity to further explore this phenomenon in the exceptional circumstances of a global health crisis. Some literature analysed what factors influenced the likelihood of dying during the pandemic, highlighting that people of older age [11–14], men [11, 12, 15, 16], and ethnic minorities [11, 12, 15–21] were disproportionately impacted by the pandemic and had a higher likelihood of dying from COVID-19. Yet, the causes of such disparities remain mixed and less explored. Existing evidence has shown that people with lower socioeconomic status were more exposed to the COVID-19 disease in the more advanced phases of the pandemic, once measures of social distancing were implemented, likely due to more crowded households and less flexible job arrangements, which might have limited the possibility of social distancing [15, 18, 22–24]. While there is a small body of literature looking at the intersection between lower socioeconomic status and older age as risk factors for COVID-19 contagion and mortality, some literature has suggested that lower health literacy, intergenerational and/or crowded households, and lower access to testing might have represented risk factors during the pandemic for people in older age and with lower socioeconomic status [25].

In addition to poorer health status and higher exposure to risk factors among people with lower socioeconomic status, disparities subsist also in healthcare utilisation at the end of life. Previous literature had highlighted that financial constraints may limit receipt of adequate long-term care and end-of-life care, particularly in the last year of life [1, 26–29]. Some recent evidence suggests that the pandemic might have amplified socioeconomic disparities in end-of-life care delivery, but existing studies are country specific, disease-specific or mostly focused on palliative care services. Evidence has in fact highlighted how the COVID-19 pandemic exacerbated socioeconomic disparities in access to palliative care among patients with cancer in Ontario (Canada) [30], to healthcare services for psychiatric patients in China [31], to end-of-life and palliative care in South-West England (United Kingdom) [32] and to specialist palliative care services for ethnic minority groups with COVID-19 in the United Kingdom (UK) [33, 34]. It has also been shown that socioeconomic gradients in receipt of palliative care across Europe increased significantly during the pandemic [35].

While palliative care is paramount to provide people at the end of life with comfort and good quality of life [36], this is not the only care service that people need and/or receive at the end of their life. The Organisation for Economic Cooperation and Development (OECD) defines end-of-life care as “*the care provided to people who are in the last 12 months of life. It refers to the terminal stage of palliative care*,* provided in the very last period of a patient’s life*,* as well as including some elements of curative care and help with mobility limitations”*^1^. Based on the OECD definition of end-of-life care, the present study aims at providing a comprehensive analysis of what type of care people received in the last year of their life before and during the COVID-19 pandemic, and which sociodemographic factors were associated with the care received.

To guide the empirical analysis, we draw on Andersen’s behavioural model of healthcare utilisation, which conceptualises the use of health services as determined by three sets of factors: predisposing factors (such as age, gender, and education), enabling factors (such as income, financial security, and access to services), and need-related factors (such as health status and morbidity) [37]. Within this framework, socioeconomic position is expected to shape end-of-life care use. Education may influence health literacy, navigation of healthcare systems, and preferences regarding care, while financial resources may affect the ability to access and sustain certain types of care, particularly in systems where coverage and availability vary across settings. These mechanisms are particularly relevant in end-of-life contexts, where care needs are complex and the coordination of services across providers and settings is often required. The COVID-19 pandemic may have further modified how these predisposing, enabling, and need-related factors translate into actual healthcare utilisation. In such crisis contexts, enabling factors such as financial resources and service availability may have become more salient, while predisposing factors such as education and health literacy may have played an increased role in navigating rapidly changing and less accessible care systems. At the same time, constraints in service provision may have limited the extent to which care was responsive to need alone.

Specifically, this study aims to examine how the receipt of end-of-life care changed before and during the COVID-19 pandemic, and whether these patterns differed across sociodemographic groups, across 28 European countries.

## Methods

### Study design and participants

The analysis is based on microdata from 5,029 decedents aged over 50, collected via proxy interviews after death, sourced from the Survey of Health, Aging and Retirement in Europe (SHARE) waves 7–9 (2017–2022) covering 28 European countries (see Table A.1 in the Appendix) [35–37]. SHARE wave 7 was collected between 2017 and 2018, wave 8 was collected between 2019 and 2020, and wave 9 was collected between 2021 and 2022 [38–40]. The end-of-life interviews were conducted with proxy respondents of SHARE participants, following the main respondent’s death. The interviews provide information on the decedent’s demographics, socioeconomic characteristics, health status, care received during the last year of life, and circumstances of their death. The study focuses on individuals who passed away between two years before and two years after the outbreak of the COVID-19 pandemic (in March 2020), for improved comparability of the sociodemographic characteristics of decedents. The sample therefore includes decedents who passed away between 2018 and 2022.

### Outcome variables

The outcome variables are six binary variables describing the type of care that the deceased received in the last year of their life, including care in hospital, in hospice, in nursing home, at home, care from a general practitioner (GP), and care from a specialist physician. The variables were built based on proxy responses in the SHARE end-of-life interview (Table A.2 in the Appendix). The definitions of these care types are based on the terminology used in the SHARE end-of-life questionnaire. While these categories are harmonized across countries within the SHARE framework, their exact interpretation may vary depending on national healthcare systems, institutional arrangements, and language-specific translations. Therefore, some degree of cross-country variation in how these care settings are defined and delivered should be expected.

### Covariates

The analysis includes covariates that describe when the person died, as well as key demographic and socio-economic characteristics of the decedents, such as gender, age, health status, partnership status, education level, financial difficulties, and country of residency:

- The variable *pandemic death* indicates whether the death took place before or after the outbreak of the COVID-19 pandemic and is categorised as before March 2020 vs. in March 2020 or later.
- The variable *gender* is categorized as male or female.
- *Age* is categorized into three age groups, 50–74 years, 75–84 years, 85 years or older.
- *Health status* is classified as poor/fair health, good health or very good/excellent health, based on self-reported information that the decedent had provided in the last interview before death.
- *Cause of death* is categorized as cancer, cardiovascular disease, COVID-19 or respiratory or infectious disease, and other cause of death. COVID-19, respiratory or infectious diseases are coded as one cause of death to include all deaths due to COVID-19, as it is possible that, particularly in the early phases of the pandemic, deaths happening due to COVID-19 were underreported due to misinterpretation of symptoms or lack of guidelines for the certification and classification of COVID-19 as a cause of death [41–43]. The variable *cause of death* is built on one question of the SHARE end-of-life interview that asks the proxy respondent to identify the decedent’s main cause of death.
- *Partnership status* is categorized as whether the deceased had a partner or not.
- The variable *education level* is classified as low (corresponding to International Standard Classification of Education (ISCED) levels 0-1-2), middle (equivalent to ISCED levels 3–4) and high (corresponding to ISCED levels 5–6) [44].
- The variable on *financial difficulty* is self-reported and classified as whether the deceased had reported their household to be able to make ends meet, in the last wave before death. The variable is categorized as making ends meet with difficulty or great difficulty vs. fairly easily or easily.
- The variable *country* refers to the country of residency where the deceased lived before death. The full list of countries included in the analysis is available in Table A.1 of Appendix A.

### Statistical analysis

First, the study examines the bivariate association between each type of care and the moment of death, using χ² tests. Next, the analysis estimates probit regression models for each outcome, including main effects and interaction terms between pandemic death and (i) cause of death, (ii) education level, and (iii) financial difficulty. Given the binary nature of the outcome variables, probit models have been used. Results were also found to be robust to alternative specifications, including linear and logit regression models a number of tests, including the Akaike Information Criterion (AIC) and the Bayesian Information Criterion (BIC) were performed to test different specifications of the model. All statistical analysis was performed using STATA (version 18.0) software. Two-sided p-values < 0.10 were considered statistically significant with outcomes presented as average marginal effects (AME) and the associated standard errors (SE).

## Results

Table 1 reports the demographic and socioeconomic characteristics of the decedents, by moment of death (before March 2020 vs. in March 2020 or later). It also reports the p-value of the chi-square test of bilateral association between each demographic and socioeconomic characteristic and the moment of death. Additionally, Tables B1-B3 in Appendix B report the distribution of care outcomes in the study population, by education level, financial difficulty and geographical area.

**Table 1:** Sociodemographic characteristics of the study population, by time of death, decedents aged 50+, *N* = 5,029

Variables	Died before the pandemic (Jan. 2018 – Feb. 2020)	Died during the pandemic (March 2020 – Dec. 2022)	Χ^2^ test
N	2,316 (46.1%)	2,713 (53.9%)	
Gender			
Male	1,237 (53.4%)	1,544 (56.9%)	0.013
Female	1,079 (46.6%)	1,169 (43.1%)	
Age at death			
50–74	673 (29.1%)	729 (26.9%)	0.192
75–84	809 (34.9%)	958 (35.3%)	
85+	834 (36.0%)	1,026 (37.8%)	
Self-rated health			
Poor/fair	1,690 (73.0%)	1,995 (73.5%)	0.617
Good	473 (20.4%)	557 (20.5%)	
Very good/excellent	153 (6.6%)	161 (5.9%)	
Main cause of death			
Cancer	702 (30.3%)	607 (22.4%)	< 0.001
Cardiovascular	848 (36.6%)	926 (34.1%)	
COVID19 or respiratory or infectious	336 (14.5%)	693 (25.5%)	
Other	430 (18.6%)	487 (18.0%)	
Partnership status			
Has a partner	1,416 (61.1%)	1,604 (59.1%)	0.145
No partner	900 (38.9%)	1,109 (40.9%)	
Education level			
Low	1,216 (52.5%)	1,355 (49.9%)	0.154
Medium	776 (33.5%)	975 (35.9%)	
High	324 (14.0%)	383 (14.1%)	
Ability to make ends meet			
(fairly) easily	1,196 (51.6%)	1,361 (50.2%)	0.297
with (great) difficulty	1,120 (48.4%)	1,352 (49.8%)	
Geographical area			
Eastern Europe	503 (21.7%)	697 (25.7%)	0.010
Northern Europe	342 (14.8%)	371 (13.7%)	
Southern Europe	971 (41.9%)	1,101 (40.6%)	
Western Europe	500 (21.6%)	544 (20.1%)	

The table shows the socioeconomic characteristics of the sample, by time of death (before March 2020 vs. in March 2020 or later), as well as the *p*-value from the chi-square tests

**Fig. 1:**
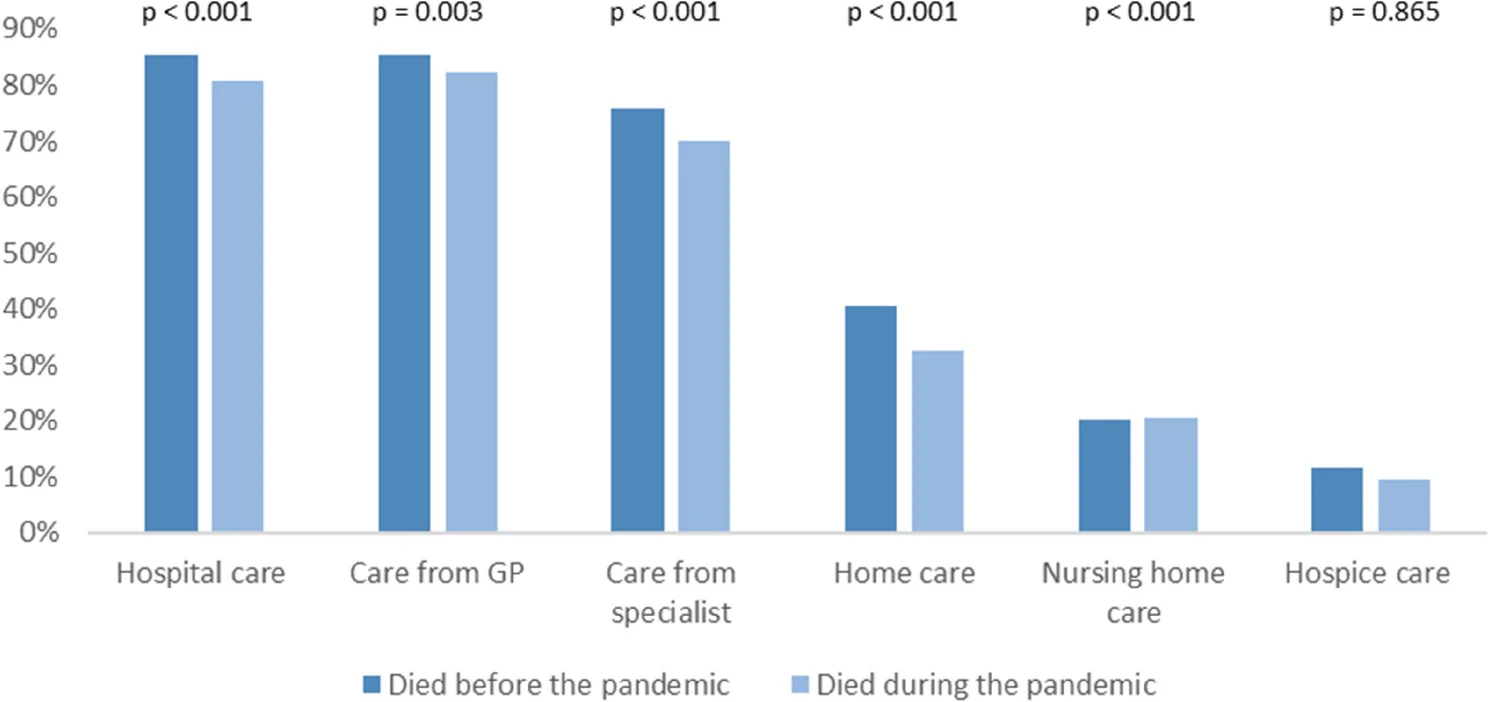
Share of decedents aged 50 + receiving care in the last year of life, before and after the beginning of the pandemic, by type of care. *N* = 5,029. Note: The figure shows the share of decedents in the sample that received care in the last year before death, by time of death and by type of care, as well as the p-value from the chi-square tests. It includes decedents who died between 2018 and 2022. “Died before the pandemic” refers to deaths between January 2018 and February 2020, “died during the pandemic” refers to deaths between March 2020 and December 2022. “GP” refers to General Practitioners

Figure 1 reports the share of decedents that received care in the last year of life, by type of care and by moment of death (before March 2020 vs. in March 2020 or later). It also reports the p-value of the chi-square test of bilateral association between each outcome variable and the moment of death.

**Fig. 2:**
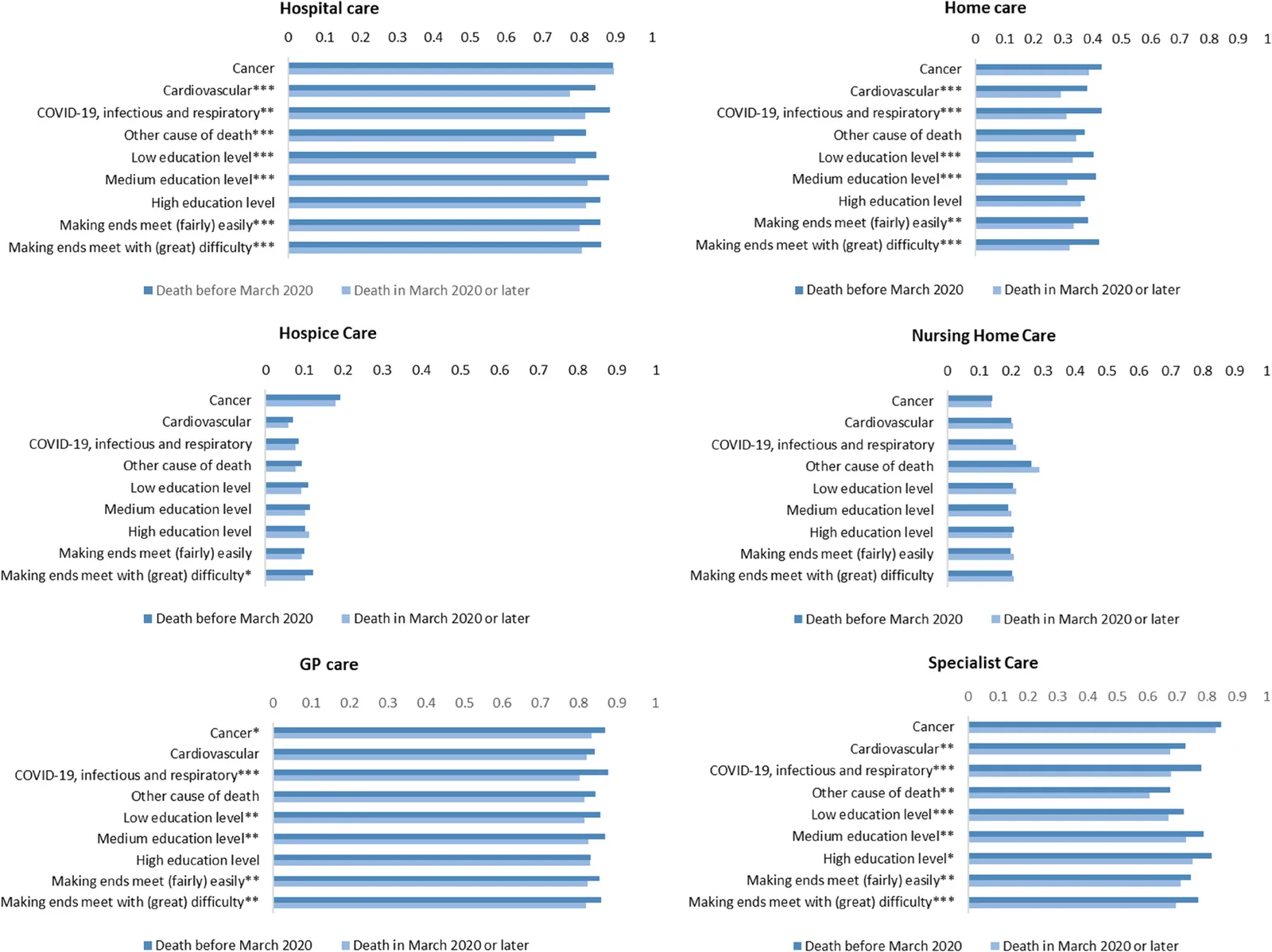
Predicted probabilities of the interaction terms of the probit regression models on the type of care received at the end of life and sociodemographic characteristics of SHARE decedents aged 50+, *n* = 5,029. Note: The figure shows predicted probabilities of the interaction terms of the probit regression models. Each figure reports the predicted probabilities of the probit regression model of each outcome variable. The models control for gender, age, health status, partnership status, education level, financial difficulties, and country. Statistical significance: * *p* < .1, ** *p* < .05, *** *p* < .001. The *p*-values for the interaction terms test the null hypothesis that the association between each predictor (cause of death, education level, financial difficulty) and the outcome does not differ by time period (before vs. during the pandemic). Statistically significant interaction terms thus indicate a differential effect of these predictors on care outcomes across the two time periods

Figure 2 reports the predicted probabilities of the interaction terms between the moment of death and (i) the cause of death, (ii) the education level, and (iii) the financial difficulty, from six binary probit regression models. These interaction terms allow us to assess whether the association between each of these factors and the type of end-of-life care received differs depending on the time period.

Table 2 reports the Average Marginal Effects of the interaction terms between the moment of death and (i) the cause of death, (ii) the education level, and (iii) the financial difficulty, from the six binary probit regression models. The models analyse the type of care that people received before dying (care in hospital, in hospice, in nursing home, at home, care from a GP, and care from a specialist physician), controlling for the moment of death, sociodemographic characteristics of the decedents and the interaction terms between the moment of death and some sociodemographic characteristics of the decedents (i.e. cause of death, education level and self-reported financial difficulty). All regression models’ coefficients are reported in Table B.1 of Appendix B. Additional robustness checks were performed, including restricting the sample to narrower time windows excluding deaths in the early months of the pandemic, expanding the sample to include a larger time window, and grouping countries by geographical regions. These analyses yielded results consistent with the main findings, and are reported in Appendix C.

**Table 2:** Average Marginal Effects of the interaction terms of probit regression models of the type of care received at the end of life and sociodemographic characteristics of SHARE decedents aged 50+, *n* = 5,029

Variables	Hospital care	Hospice care	Nursing home care	Home care	GP care	Specialist physician care
Death in March 2020 or later#cancer death	0.001	-0.009	-0.003	-0.041	-0.036*	-0.018
	(0.016)	(0.021)	(0.017)	(0.026)	(0.020)	(0.019)
Death in March 2020 or later#cardiovascular death	-0.070***	-0.013	0.005	-0.094***	-0.022	-0.051**
	(0.018)	(0.012)	(0.018)	(0.021)	(0.017)	(0.021)
Death in March 2020 or later#COVID-19, respiratory and infectious death	-0.070**	-0.009	0.008	-0.123***	-0.075***	-0.101***
	(0.023)	(0.017)	(0.024)	(0.031)	(0.022)	(0.027)
Death in March 2020 or later#other death	-0.090***	-0.018	0.029	-0.027	-0.026	-0.065**
	(0.028)	(0.019)	(0.030)	(0.031)	(0.024)	(0.031)
Death in March 2020 or later#low education	-0.062***	-0.020*	0.010	-0.081***	-0.042**	-0.057***
	(0.015)	(0.011)	(0.015)	(0.019)	(0.014)	(0.018)
Death in March 2020 or later#medium education	-0.053***	-0.011	0.010	0.094***	-0.045**	-0.054**
	(0.015)	(0.015)	(0.017)	(0.022)	(0.017)	(0.019)
Death in March 2020 or later#high education	-0.035	0.012	-0.005	-0.001	0.002	-0.054*
	(0.026)	(0.023)	(0.029)	(0.035)	(0.028)	(0.029)
Death in March 2020 or later#make ends meet (fairly) easily	-0.055***	-0.004	0.011	-0.044**	-0.029**	-0.038**
	(0.014)	(0.012)	(0.016)	(0.018)	(0.014)	(0.017)
Death in March 2020 or later#with (great) difficulty	-0.056***	-0.021*	0.005	-0.105***	-0.046**	-0.073***
	(0.015)	(0.012)	(0.014)	(0.019)	(0.015)	(0.017)

The table shows average marginal effects and standard errors in parentheses, of the interaction terms of the probit regression models. Each column reports the results of the probit regression model of each outcome variable. The models control for gender, age, health status, partnership status, education level, financial difficulties, and country

Statistical significance: * *p* < .1, ** *p* < .05, *** *p* < .001

The recipience of end-of-life care decreased overall for all decedents after the outbreak of the pandemic in March 2020. Nevertheless, results show an association between the shift in the type of care that people received in their last year of life after the outbreak of the pandemic and their cause of death, education level and financial difficulty.

After the beginning of the pandemic, the shift in probabilities of receiving the different types of care under analysis varied substantially by cause of death. For decedents dying of cardiovascular causes, the average marginal effect (AME) associated with the pandemic period indicates a significant 7.0%-point lower probability of hospital care utilization (AME = − 0.070, *p* < .001) and a concomitant 9.4-point lower probability of receiving home care (AME = − 0.094, *p* < .001), alongside a smaller but significant 5.1-point lower probability to receive specialist physician care (AME = − 0.051, *p* < .01). Dying of COVID-19 or other respiratory/infectious was associated with a similarly sized 7.0-point lower hospital care use (AME = − 0.070, *p* < .01), a 12.3-point lower home care use (AME = − 0.123, *p* < .001), a 7.5-point lower probability to receive care from a GP (AME = − 0.075, *p* < .001), and a 10.1-point lower probability to receive specialist care (AME = − 0.101, *p* < .001). For other causes of death, the AME associated with the pandemic period was a 9.0-point lower probability to receive hospital care (AME = − 0.090, *p* < .001) and a 6.5-point lower specialist care use (AME = − 0.065, *p* < .01). In contrast, cancer deaths showed no significant changes across hospital, hospice, nursing home, or home care, though a modest 3.6-point lower receipt of care from GP reached marginal significance (AME = − 0.036, *p* < .05). Overall, non-cancer causes of death were associated with lower probabilities of receiving both institutional and home-based services after the outbreak of the pandemic, highlighting a marked shift in end-of-life care trajectories by cause of death.

Associations between the pandemic period and care settings differed by educational attainment in nuanced ways. Low education levels were associated with 6.2 point lower hospital care use (AME = − 0.062, *p* < .001), a 2.0 point lower hospice utilization (AME = − 0.020, *p* < .05), an 8.1 point lower home care use (AME = − 0.081, *p* < .001), a 4.2 point lower probability to receive care from a GP (AME = − 0.042, *p* < .01), and a 5.7 point lower probability of specialist visits (AME = − 0.057, *p* < .001). Medium education levels were similarly associated with a 5.3 point lower hospital care (AME = − 0.053, *p* < .001), a 5.4 point lower specialist care use (AME = − 0.054, *p* < .01) and a 4.5 point lower probability to receive care form a GP (AME = − 0.045, *p* < .01), but were also associated with a significant 9.4 point higher home care use (AME = + 0.094, *p* < .001). In contrast, the high education group showed no significant AMEs for hospital, hospice, nursing home, or home care; only a modest 5.4 point lower specialist care use reached marginal significance (AME = − 0.054, *p* < .05). These patterns suggest that lower levels of institutional care use during the pandemic were most pronounced among lower educated decedents, while medium educated individuals partially shifted toward increased home-based support.

Financial circumstances before death are strongly associated with changes in the care received in the last year of life during the pandemic. Reporting making ends meet easily or fairly easily was associated with a 5.5-point lower hospital care use (AME = − 0.055, *p* < .001), a 4.4-point lower home care use (AME = − 0.044, *p* < .01), a 2.9-point lower GP utilization (AME = − 0.029, *p* < .01), and a 3.8-point lower specialist care receipt (AME = − 0.038, *p* < .01). Reporting difficulty or great difficulty was associated with a nearly identical 5.6-point lower hospital care utilization (AME = − 0.056, *p* < .001), a 10.5-point lower probability of receiving home care (AME = − 0.105, *p* < .001), a 4.6-point lower probability of GP visits (AME = − 0.046, *p* < .01), and a 7.3-point lower specialist care use (AME = − 0.073, *p* < .001), alongside a 2.1-point lower hospice care use (AME = − 0.021, *p* < .05). These results suggest that financially strained decedents faced the steepest cutbacks in both institutional and home-based care, particularly in home care utilization, underscoring widening socioeconomic disparities in end-of-life services following the pandemic.

## Discussion

The analysis found lower levels of care among older Europeans who died after the outbreak of the pandemic. This holds for all types of end-of-life care, except for nursing home care. Such decrease ranged from a 20% decrease in home care to almost 4% decrease for care from a GP. These results are in line with previous analysis showing that older people who were receiving care at home prior to the pandemic were heavily affected by the shortage of care services during the crisis [13, 45]. Furthermore, in most OECD countries, including European countries, day care centres were closed during the pandemic. People living and receiving care at home and visiting day care centres might have therefore experienced a greater decrease of care receipt during the pandemic [13, 45]. The steady use of care in nursing homes might result from the fact that during the pandemic nursing homes had reduced the transfer of patient from and to the facilities, to avoid the spread of COVID-19 through movement of people across settings of care [45, 46].

While care use decreased overall, this analysis also found that older Europeans who died during the COVID-19 pandemic received different types of care at the end of their life across socioeconomic groups. The type of care that decedents received was associated with factors such as their cause of death, education level and the financial circumstances. It is noteworthy that existing research has documented shifts in the socio-demographic profiles of decedents during the pandemic [47], suggesting that differences in end-of-life care across periods may also partly reflect changes in the composition of decedents before and after the pandemic.

### Cause of death

Dying due to cardiovascular diseases was associated with a greater decrease in care use after the outbreak of the pandemic, with a significant reduction in receipt of home care, hospital care and care from a specialist. This result is in line with previous literature highlighting that the pandemic heavily disrupted cardiovascular care, including primary and secondary prevention, elective procedures and acute care for cardiovascular diseases [48–50]. Part of the reduction in cardiovascular care has been attributed to medical care avoidance and isolation which might have caused underdiagnosis due to early symptoms going unnoticed. Another reason for decreased cardiovascular care during the pandemic has been suggested to be linked to a need of adaptation of health systems, with reduced admissions or deferrals of less urgent cases, due to limited capacity of the heath system during the pandemic [51]. On the other end, dying due to cancer was associated with the smallest changes in care receipt, with only a statistically significant lower probability of GP care by 3.6% point. While existing literature highlighted cancer care disruption during the pandemic, particularly for screening and care plans [52, 53], our findings show small changes in care receipt associated with cancer deaths. Such findings, while not implying any causal relation due to the nature of the study design, might suggest that early cancer care was more impacted by the pandemic compared to care for people towards the end of life.

### Education level

Education level also seemed to be associated to changes in care use, although the pattern is mixed. High educational attainment was associated with only slightly lower care received from a specialist physician during the pandemic, while people with low education level experienced lower probability of receiving all types of care, except for nursing home care. The lower probability of care use for people with lower education level ranged between 8.1% for home care, to 2.0% for hospice care. One possible explanation for the lower receipt of specialist physician care might be an effort to obey to non-pharmaceutical measures, such as social distancing, to minimize the risk of COVID-19 contagion. Existing evidence has in fact highlighted that people with higher education were more likely to comply with non-pharmaceutical measures against contagion [20, 54–56]. People with medium education level experienced mixed associations of the pandemic on the care received at the end of life, with a shift away from hospital care, care from a GP and from a specialist physician, towards more care at home.

### Financial circumstances

The financial circumstances that decedents had declared before death are associated with the care received at the end of life, uncovering potential economic disparities in end-of-life care use. Reporting making ends meet with difficulty or great difficulty was associated with lower care receipt in all settings, with the exception of care in nursing homes, which remained unchanged. The greatest decrease was in outpatient care (i.e. -10.5% in home care, -7.3% in specialist physician care), but some decrease also concerned inpatient care, such as hospital care (− 5.6%) and hospice care (-2.1%). Reporting making ends meet easily or fairly easily was associated with slightly lower care use after the outbreak of the pandemic, ranging from − 2.9% for GP care, to -5.5% for hospital care. It is noteworthy that the economic dividend in care use appears to be widest for home care (around 4% points) and the narrowest for hospital care (0.1% points). Previous analysis revealed that public funding for end-of-life care is geared towards hospital settings, while public funding for home care is less common across OECD countries 1. The results of our analysis might suggest that care cost might represent a barrier to access for people with lower financial stability, although deeper analysis is needed, to explore the effect of increased public funding for end-of-life care on the care received in the last year of life.

## Limitations

For instance, information on the cause of death is reported by proxy respondents, which might introduce biases and misclassification particularly for COVID-19 deaths, for which exact classification at the beginning of the pandemic was not always possible. To mitigate this bias, we have classified COVID-19 deaths by including deaths for which COVID-19 was reported as the main cause of death, as well as deaths due to infectious and respiratory diseases. Furthermore, since end-of-life interviews rely on the availability of a proxy, individuals without close relatives may be underrepresented in the sample. Furthermore, the lack of systematic linkage between SHARE data and mortality registers across all participating countries does not allow to determine the status of respondents for whom no end-of-life interview is available.

Due to the survey design, the outcome variables refer to care received in the last year of life, while the pandemic indicator is defined based on the timing of death. As a result, individuals who died shortly after the onset of the pandemic may have received a substantial portion of their care before March 2020. This may introduce some measurement error in the analysis. The associations in the study are therefore likely to be underestimated, and results are likely to be conservative.

Although the study takes geographical variations into account, the pooling of data from 28 countries limits the ability to draw detailed conclusions regarding country-specific cultural and socioeconomic factors. Additionally, the survey design results in pandemic decedents being slightly older than those from the pre-pandemic period. However, performing an age-stratified analysis we obtained very similar results, which supports the robustness of the results.

Another limitation relates to the non-random selection of individuals into different healthcare settings. Care use can be influenced by socioeconomic characteristics, health conditions, family support, and institutional factors. Although the analysis controls for several observed determinants of care utilisation, including age, health status, cause of death, partnership status, and socioeconomic characteristics, unobserved factors may still affect both the likelihood of receiving certain types of care and the outcomes observed.

Despite these limitations, the rich end-of-life data provided by SHARE offer a valuable and unique opportunity to explore how end-of-life care provision has changed after the beginning of the pandemic, across sociodemographic groups.

## Conclusion

Lower levels of end-of-life care use were observed among individuals who died during the pandemic period. Our analysis suggests that gradients in the receipt of healthcare services in the last year of life might have widened during the pandemic. Greater decreases in end-of-life care receipt after March 2020 were associated with deaths due to cardiovascular diseases, lower education and difficulty making ends meet. Care at home showed the greatest changes during the pandemic.

According to existing frameworks of care utilization such as Andersen’s behavioural model of care utilization [37], the use of healthcare services is shaped by a combination of predisposing factors (e.g. age, education), enabling factors (e.g. financial resources), and need factors (e.g. health status). Differences in the receipt of end-of-life care across sociodemographic groups therefore likely reflect a complex interplay of structural, cultural, and individual-level factors. Structural barriers may include financial constraints, unequal geographical distribution of services, and limited accessibility of care facilities. Navigating end-of-life care can be particularly challenging, especially for individuals with multimorbidity who must interact with multiple providers within systems that are not always well coordinated or integrated. Cultural and linguistic barriers, as well as differences in health literacy and personal preferences regarding care, may further contribute to disparities in the type and setting of care received [1, 57].

These findings highlight the need for policies that strengthen the accessibility, coordination, and equity of end-of-life care. In particular, reinforcing home-based care services, reducing financial barriers to access, and improving the integration of care across providers could help ensure that individuals receive care aligned with their needs and preferences.

Further research is needed to better understand the root causes of disparities in end-of-life care across socioeconomic groups. In particular, examining how individual characteristics interact with structural features of healthcare systems—such as funding mechanisms, accessibility, quality, and people-centredness—would support more effective and evidence-based policy interventions aimed at ensuring equitable access to high-quality care at the end of life.

## Supplementary Information

Supplementary Material 1.

## Data Availability

The data that support the findings of this study are available from SHARE-ERIC (2024). Survey of Health, Ageing and Retirement in Europe (SHARE) Wave 1-9. Release version: 9.0.0. SHARE-ERIC, but restrictions apply to the availability of these data, which were used under license for the current study, and so are not publicly available. Data are however available from the authors upon reasonable request and with permission of SHARE-ERIC.
