# Supplementary material for: Unequal goodbyes: disparities in end-of-life care before and after the outbreak of the COVID-19 pandemic across 28 countries
Source: BMC Palliat Care. 2026 Jul 7;25:268. doi: 10.1186/s12904-026-02220-y (PMC13560249; doi:10.1186/s12904-026-02220-y)
Supplement: Supplementary file 1 [file 12904_2026_2220_MOESM1_ESM.docx]

**Appendix A. Methodology**

**Table A.1 Observations by country**

| **Countries** | **n (2018-2022)** | **n (March 2020- 2022)** |
| --- | --- | --- |
| Austria | 254 | 143 |
| Belgium | 247 | 134 |
| Bulgaria | 82 | 46 |
| Croatia | 216 | 132 |
| Cyprus | 51 | 23 |
| Czech Republic | 303 | 168 |
| Denmark | 150 | 63 |
| Estonia | 489 | 253 |
| Finland | 33 | 23 |
| France | 132 | 66 |
| Germany | 177 | 95 |
| Greece | 256 | 126 |
| Hungary | 189 | 111 |
| Israel | 121 | 64 |
| Italy | 313 | 171 |
| Latvia | 97 | 71 |
| Lithuania | 132 | 65 |
| Malta | 50 | 30 |
| Netherlands | 103 | 41 |
| Poland | 414 | 258 |
| Portugal | 88 | 44 |
| Romania | 168 | 96 |
| Slovenia | 321 | 172 |
| Spain | 217 | 116 |
| Sweden | 251 | 119 |
| Switzerland | 105 | 51 |
| Luxembourg | 26 | 14 |
| Slovakia | 44 | 18 |
|  |  |  |
| Total | 5,029 | 2,713 |

*The table reports the number of observations by country for the whole sample (decedents who died between 2018 and 2022) and for decedents who died during the pandemic (from March 2020 to 2022).*

**Table A.2. SHARE end-of-life interview question used to build the outcome variables**

| **Question asked to proxy respondent to identify the care that the decedent received during their last year of life** | |
| --- | --- |
| XT018_ | Did [{Name of the deceased}] have any [care from a general practitioner/ care from specialist physicians/ hospital stays/ care in a nursing home/ hospice stays/ medication/ aids and appliances such as wheelchairs, rollators, walking sticks and crutches, orthoses, or protheses/ help with personal care due to disability/ help with domestic tasks due to disability] (in the last 12 months of[his/ her] life)?[Help \"with personal care due to disability\" refers here to professional help/care/assistance received at home (not in a healthcare facility)./ Help \"with domestic tasks due to disability\" refers here to professional help/care/assistance received at home (not in a healthcare facility).]  1. Yes  5. No |

**Appendix B. Results**

**Table B.1. Distribution of care outcomes in the study population, by education level, decedents aged 50+, N= 5,029**

|  | **education level** | | | |
| --- | --- | --- | --- | --- |
|  | **low** | **medium** | **high** | **Χ^2^ test** |
| **N** | 2,571 (51.1%) | 1,751 (34.8%) | 707 (14.1%) |  |
| **Hospital care** | 2,056 (80.0%) | 1,519 (86.8%) | 596 (84.3%) | <0.001 |
| **Nursing home care** | 2,003 (77.9%) | 1,445 (82.5%) | 553 (78.2%) | <0.001 |
| **Hospice care** | 249 (9.7%) | 187 (10.7%) | 79 (11.2%) | 0.387 |
| **Home care** | 972 (37.8%) | 583 (33.3%) | 265 (37.5%) | 0.008 |
| **GP care** | 2,161 (84.1%) | 1,461 (83.4%) | 583 (82.5%) | 0.581 |
| **Specialist care** | 1,757 (68.3%) | 1,345 (76.8%) | 550 (77.8%) | <0.001 |
|  |  |  |  |  |

**Table B.2. Distribution of care outcomes in the study population, by financial difficulty, decedents aged 50+, N= 5,029**

|  | **Is household able to make ends meet** | | |
| --- | --- | --- | --- |
|  | **(fairly) easily** | **with (great) difficulty** | **Χ^2^ test** |
| **N** | 2,557 (50.8%) | 2,472 (49.2%) |  |
| **Hospital care** | 2,123 (83.0%) | 2,048 (82.8%) | 0.866 |
| **Nursing home care** | 622 (24.3%) | 406 (16.4%) | <0.001 |
| **Hospice care** | 278 (10.9%) | 237 (9.6%) | 0.133 |
| **Home care** | 988 (38.6%) | 832 (33.7%) | <0.001 |
| **GP care** | 2,151 (84.1%) | 2,054 (83.1%) | 0.323 |
| **Specialist care** | 1,846 (72.2%) | 1,806 (73.1%) | 0.492 |
|  |  |  |  |

**Table B.3. Distribution of care outcomes in the study population, by geographical area, decedents aged 50+, N= 5,029**

|  | **Geographical area** | | | | |
| --- | --- | --- | --- | --- | --- |
|  | **Eastern Europe** | **Northern Europe** | **Southern Europe** | **Western Europe** | **Χ2 test** |
| **N** | 1,200 (23.9%) | 713 (14.2%) | 2,072 (41.2%) | 1,044 (20.8%) |  |
| **Hospital care** | 1,064 (88.7%) | 595 (83.5%) | 1,646 (79.4%) | 866 (83.0%) | <0.001 |
| **Nursing home care** | 128 (10.7%) | 192 (26.9%) | 351 (16.9%) | 357 (34.2%) | <0.001 |
| **Hospice care** | 77 (6.4%) | 92 (12.9%) | 203 (9.8%) | 143 (13.7%) | <0.001 |
| **Home care** | 352 (29.3%) | 277 (38.8%) | 716 (34.6%) | 475 (45.5%) | <0.001 |
| **GP care** | 1,041 (86.8%) | 550 (77.1%) | 1,674 (80.8%) | 940 (90.0%) | <0.001 |
| **Specialist care** | 989 (82.4%) | 442 (62.0%) | 1,449 (69.9%) | 772 (73.9%) | <0.001 |
|  |  |  |  |  |  |

**Table B.4. Coefficients of probit regression models of the type of care received at the end of life and sociodemographic characteristics of SHARE decedents aged 50+, n = 5,029**

| **Variables** | **Hospital care** | **Hospice care** | **Nursing home care** | **Home care** | **GP care** | **Specialist physician care** |
| --- | --- | --- | --- | --- | --- | --- |
| Time of death (Death before March 2020)  Death in March 2020 or later | 0.009 | -0.047 | 0.002 | -0.046 | -0.161 | 0.002 |
|  | (0.120) | (0.116) | (0.119) | (0.095) | (0.113) | (0.108) |
| Cause of death (Cancer)  Cardiovascular | -0.247*** | -0.626*** | 0.271*** | -0.139** | -0.124 | -0.460*** |
|  | (0.088) | (0.092) | (0.088) | (0.069) | (0.083) | (0.080) |
| COVID19 or respiratory or infectious | -0.048 | -0.528*** | 0.297*** | -0.003 | 0.047 | -0.277*** |
|  | (0.115) | (0.121) | (0.109) | (0.088) | (0.113) | (0.102) |
| Other | -0.358*** | -0.475*** | 0.513*** | -0.160* | -0.121 | -0.618*** |
|  | (0.100) | (0.106) | (0.096) | (0.084) | (0.101) | (0.092) |
| Death before March 2020 # Cancer | 0.000 | 0.000 | 0.000 | 0.000 | 0.000 | 0.000 |
|  | (0.000) | (0.000) | (0.000) | (0.000) | (0.000) | (0.000) |
| Death before March 2020 # Cardiovascular diseases | 0.000 | 0.000 | 0.000 | 0.000 | 0.000 | 0.000 |
|  | (0.000) | (0.000) | (0.000) | (0.000) | (0.000) | (0.000) |
| Death before March 2020 # COVID-19/respiratory/infectious disease | 0.000 | 0.000 | 0.000 | 0.000 | 0.000 | 0.000 |
|  | (0.000) | (0.000) | (0.000) | (0.000) | (0.000) | (0.000) |
| Death before March 2020 # Other cause of death | 0.000 | 0.000 | 0.000 | 0.000 | 0.000 | 0.000 |
|  | (0.000) | (0.000) | (0.000) | (0.000) | (0.000) | (0.000) |
| Death in March 2020 or later# Cancer | 0.000 | 0.000 | 0.000 | 0.000 | 0.000 | 0.000 |
|  | (0.000) | (0.000) | (0.000) | (0.000) | (0.000) | (0.000) |
| Death in March 2020 or later# Cardiovascular diseases | -0.280** | -0.056 | 0.038 | -0.150 | 0.073 | -0.065 |
|  | (0.123) | (0.129) | (0.123) | (0.098) | (0.114) | (0.110) |
| Death in March 2020 or later# COVID-19/respiratory/infectious disease | -0.321** | -0.013 | 0.050 | -0.228** | -0.173 | -0.248* |
|  | (0.147) | (0.155) | (0.143) | (0.115) | (0.140) | (0.130) |
| Death in March 2020 or later# Other cause of death | -0.310** | -0.066 | 0.101 | 0.029 | 0.042 | -0.112 |
|  | (0.138) | (0.147) | (0.134) | (0.115) | (0.136) | (0.125) |
| Education level (low)  Medium | 0.178** | 0.021 | -0.069 | 0.022 | 0.064 | 0.236*** |
|  | (0.080) | (0.084) | (0.076) | (0.064) | (0.077) | (0.070) |
| High | 0.051 | -0.042 | 0.006 | -0.086 | -0.110 | 0.336*** |
|  | (0.104) | (0.115) | (0.099) | (0.086) | (0.100) | (0.097) |
| Death before March 2020 # Low education | 0.000 | 0.000 | 0.000 | 0.000 | 0.000 | 0.000 |
|  | (0.000) | (0.000) | (0.000) | (0.000) | (0.000) | (0.000) |
| Death before March 2020 # Medium education | 0.000 | 0.000 | 0.000 | 0.000 | 0.000 | 0.000 |
|  | (0.000) | (0.000) | (0.000) | (0.000) | (0.000) | (0.000) |
| Death before March 2020 # High education | 0.000 | 0.000 | 0.000 | 0.000 | 0.000 | 0.000 |
|  | (0.000) | (0.000) | (0.000) | (0.000) | (0.000) | (0.000) |
| Death in March 2020 or later # Low education | 0.000 | 0.000 | 0.000 | 0.000 | 0.000 | 0.000 |
|  | (0.000) | (0.000) | (0.000) | (0.000) | (0.000) | (0.000) |
| Death in March 2020 or later # Medium education | -0.051 | 0.049 | 0.008 | -0.080 | -0.019 | -0.049 |
|  | (0.101) | (0.113) | (0.099) | (0.084) | (0.099) | (0.090) |
| Death in March 2020 or later # High education | 0.061 | 0.168 | -0.061 | 0.169 | 0.177 | -0.074 |
|  | (0.136) | (0.153) | (0.132) | (0.116) | (0.133) | (0.126) |
| Ability to make ends meet (easily or fairly easily)  with (great) difficulty | 0.017 | 0.135* | 0.021 | 0.111* | 0.022 | 0.078 |
|  | (0.073) | (0.079) | (0.071) | (0.060) | (0.072) | (0.065) |
| Death before March 2020 # Make ends meet (fairly) easily | 0.000 | 0.000 | 0.000 | 0.000 | 0.000 | 0.000 |
|  | (0.000) | (0.000) | (0.000) | (0.000) | (0.000) | (0.000) |
| Death before March 2020 # Make ends meet (fairly) easily | 0.000 | 0.000 | 0.000 | 0.000 | 0.000 | 0.000 |
|  | (0.000) | (0.000) | (0.000) | (0.000) | (0.000) | (0.000) |
| Death in March 2020 or later # Make ends meet with (great) difficulty | 0.000 | 0.000 | 0.000 | 0.000 | 0.000 | 0.000 |
|  | (0.000) | (0.000) | (0.000) | (0.000) | (0.000) | (0.000) |
| Death in March 2020 or later # Make ends meet with (great) difficulty | 0.006 | -0.088 | -0.023 | -0.153** | -0.040 | -0.127 |
|  | (0.091) | (0.104) | (0.091) | (0.078) | (0.091) | (0.082) |
| Gender (Male)  Female | -0.128*** | 0.071 | 0.150*** | 0.097** | -0.046 | -0.022 |
|  | (0.048) | (0.056) | (0.048) | (0.041) | (0.048) | (0.044) |
| Age at death (50-74)  75-84 | -0.062 | 0.061 | 0.283*** | 0.185*** | 0.098* | -0.031 |
|  | (0.061) | (0.066) | (0.063) | (0.050) | (0.057) | (0.054) |
| 85+ | -0.203*** | 0.027 | 0.563*** | 0.386*** | 0.086 | -0.289*** |
|  | (0.063) | (0.072) | (0.064) | (0.054) | (0.061) | (0.056) |
| Self-rated health (poor/fair)  good | -0.047 | 0.029 | -0.138** | -0.240*** | -0.115** | -0.109** |
|  | (0.056) | (0.065) | (0.058) | (0.050) | (0.056) | (0.051) |
| very good/excellent | 0.030 | 0.125 | -0.118 | -0.265*** | -0.354*** | -0.173** |
|  | (0.096) | (0.104) | (0.099) | (0.083) | (0.087) | (0.086) |
| Partnership status (has a partner)  No partner | -0.023 | 0.041 | 0.281*** | 0.013 | 0.175*** | -0.012 |
|  | (0.050) | (0.059) | (0.050) | (0.044) | (0.051) | (0.046) |
| Country (Austria)  Germany | 0.505*** | 0.254 | 0.089 | 0.804*** | 0.250 | 0.386*** |
|  | (0.184) | (0.196) | (0.137) | (0.128) | (0.180) | (0.142) |
| Sweden | -0.207 | 0.666*** | 0.017 | -0.184 | -0.354** | -0.083 |
|  | (0.132) | (0.171) | (0.123) | (0.122) | (0.141) | (0.118) |
| Netherlands | -0.361** | 0.413* | 0.286* | 0.499*** | -0.193 | 0.468*** |
|  | (0.172) | (0.216) | (0.163) | (0.152) | (0.185) | (0.174) |
| Spain | 0.143 | 0.489*** | -0.431*** | 0.507*** | 0.182 | 0.518*** |
|  | (0.147) | (0.184) | (0.138) | (0.123) | (0.167) | (0.132) |
| Italy | -0.067 | 0.220 | -0.577*** | 0.144 | -0.018 | 0.190 |
|  | (0.132) | (0.179) | (0.132) | (0.115) | (0.145) | (0.117) |
| France | 0.185 | 0.978*** | 0.246* | 0.769*** | 0.241 | 0.416*** |
|  | (0.174) | (0.186) | (0.146) | (0.141) | (0.200) | (0.152) |
| Denmark | -0.221 | 0.300 | 0.402*** | 0.975*** | -0.775*** | -0.528*** |
|  | (0.154) | (0.200) | (0.141) | (0.137) | (0.152) | (0.135) |
| Greece | -0.438*** | -1.026*** | -1.616*** | -0.127 | -0.451*** | 0.338*** |
|  | (0.135) | (0.368) | (0.211) | (0.126) | (0.145) | (0.127) |
| Switzerland | -0.441*** | 1.016*** | 0.474*** | 0.802*** | -0.337* | -0.195 |
|  | (0.164) | (0.195) | (0.154) | (0.151) | (0.178) | (0.152) |
| Belgium | -0.145 | 0.609*** | 0.366*** | 0.351*** | 0.421** | 0.280** |
|  | (0.135) | (0.173) | (0.122) | (0.118) | (0.171) | (0.122) |
| Israel | 0.139 | -0.321 | -0.633*** | 0.778*** | -0.263 | -0.028 |
|  | (0.177) | (0.289) | (0.180) | (0.146) | (0.175) | (0.149) |
| Czech Republic | 0.100 | -0.012 | -0.419*** | 0.356*** | 0.018 | 0.872*** |
|  | (0.133) | (0.189) | (0.125) | (0.112) | (0.144) | (0.130) |
| Poland | 0.556*** | 0.253 | -0.953*** | 0.118 | -0.084 | 0.223** |
|  | (0.142) | (0.171) | (0.139) | (0.110) | (0.135) | (0.111) |
| Luxembourg | 0.160 | 0.757** | 0.051 | -0.331 | -0.517* | 1.362*** |
|  | (0.342) | (0.326) | (0.291) | (0.299) | (0.294) | (0.492) |
| Hungary | 0.039 | 0.314 | -0.184 | -0.418*** | -0.162 | 0.636*** |
|  | (0.152) | (0.196) | (0.140) | (0.142) | (0.159) | (0.144) |
| Portugal | -0.486*** | 0.973*** | -0.195 | 0.932*** | -0.532*** | -0.147 |
|  | (0.177) | (0.207) | (0.183) | (0.165) | (0.185) | (0.167) |
| Slovenia | -0.419*** | 0.147 | -0.101 | -0.194* | -0.849*** | -0.166 |
|  | (0.125) | (0.179) | (0.120) | (0.117) | (0.131) | (0.112) |
| Estonia | -0.152 | 0.536*** | -0.102 | 0.007 | -0.493*** | -0.135 |
|  | (0.119) | (0.160) | (0.110) | (0.105) | (0.126) | (0.104) |
| Croatia | -0.039 | 0.613*** | -0.468*** | -0.077 | -0.112 | 0.427*** |
|  | (0.146) | (0.179) | (0.148) | (0.130) | (0.153) | (0.134) |
| Lithuania | 0.226 | 0.439** | 0.002 | 0.186 | -0.256 | 0.215 |
|  | (0.181) | (0.205) | (0.150) | (0.143) | (0.173) | (0.150) |
| Bulgaria | -0.254 | 0.118 | -1.501*** | -0.734*** | -0.238 | 0.200 |
|  | (0.188) | (0.265) | (0.388) | (0.222) | (0.200) | (0.178) |
| Cyprus | -0.267 | 0.517* | -0.543** | 0.018 | -0.233 | 0.119 |
|  | (0.218) | (0.281) | (0.238) | (0.204) | (0.241) | (0.207) |
| Finland | 0.899* | 0.723** | 0.025 | 0.619*** | -0.772*** | 0.188 |
|  | (0.481) | (0.295) | (0.264) | (0.238) | (0.252) | (0.262) |
| Latvia | 0.402* | 0.351 | -0.630*** | 0.414*** | -0.439** | -0.247 |
|  | (0.214) | (0.231) | (0.199) | (0.159) | (0.183) | (0.159) |
| Malta | -0.163 | 0.311 | -0.945*** | -0.330 | -0.675*** | -0.555*** |
|  | (0.229) | (0.292) | (0.304) | (0.226) | (0.216) | (0.201) |
| Romania | 0.092 | 0.029 | -0.914*** | 0.010 | -0.280* | 0.481*** |
|  | (0.162) | (0.218) | (0.187) | (0.138) | (0.160) | (0.147) |
| Slovakia | 0.007 | -^[1]^ | -0.062 | 0.404* | 0.188 | 0.877*** |
|  | (0.274) |  | (0.257) | (0.215) | (0.294) | (0.312) |
| Constant | 1.434*** | -1.393*** | -1.380*** | -0.601*** | 1.311*** | 0.916*** |
|  | (0.137) | (0.170) | (0.132) | (0.116) | (0.142) | (0.121) |
|  |  |  |  |  |  |  |
| Observations | 5,029 | 4,985 | 5,029 | 5,029 | 5,029 | 5,029 |
|  |  |  |  |  |  |  |

**Appendix C. Robustness checks**

**Table C.1. Coefficients of probit regression models of the type of care received at the end of life and sociodemographic characteristics of SHARE decedents aged 50+, n = 6,418, with extended time period (2016-2022)**

| VARIABLES | **Hospital care** | **Hospice care** | **Nursing home care** | **Home care** | **GP care** | **Specialist physician care** |
| --- | --- | --- | --- | --- | --- | --- |
|  |  |  |  |  |  |  |
| Time of death (Death before March 2020)  Death in March 2020 or later | -0.036 | -0.092 | 0.064 | -0.057 | -0.122 | -0.025 |
|  | (0.108) | (0.105) | (0.107) | (0.085) | (0.100) | (0.097) |
| Cause of death (Cancer)  Cardiovascular | -0.175** | -0.637*** | 0.350*** | -0.093* | -0.083 | -0.380*** |
|  | (0.069) | (0.073) | (0.070) | (0.054) | (0.066) | (0.062) |
| COVID19 or respiratory or infectious | -0.009 | -0.477*** | 0.397*** | 0.067 | 0.089 | -0.263*** |
|  | (0.092) | (0.094) | (0.087) | (0.070) | (0.090) | (0.080) |
| Other | -0.344*** | -0.477*** | 0.691*** | -0.090 | -0.060 | -0.556*** |
|  | (0.079) | (0.084) | (0.076) | (0.065) | (0.080) | (0.072) |
| Death before March 2020 # Cancer | 0.000 | 0.000 | 0.000 | 0.000 | 0.000 | 0.000 |
|  | (0.000) | (0.000) | (0.000) | (0.000) | (0.000) | (0.000) |
| Death before March 2020 # Cardiovascular diseases | 0.000 | 0.000 | 0.000 | 0.000 | 0.000 | 0.000 |
|  | (0.000) | (0.000) | (0.000) | (0.000) | (0.000) | (0.000) |
| Death before March 2020 # COVID-19/respiratory/infectious disease | 0.000 | 0.000 | 0.000 | 0.000 | 0.000 | 0.000 |
|  | (0.000) | (0.000) | (0.000) | (0.000) | (0.000) | (0.000) |
| Death before March 2020 # Other cause of death | 0.000 | 0.000 | 0.000 | 0.000 | 0.000 | 0.000 |
|  | (0.000) | (0.000) | (0.000) | (0.000) | (0.000) | (0.000) |
| Death in March 2020 or later# Cancer | 0.000 | 0.000 | 0.000 | 0.000 | 0.000 | 0.000 |
|  | (0.000) | (0.000) | (0.000) | (0.000) | (0.000) | (0.000) |
| Death in March 2020 or later# Cardiovascular diseases | -0.355*** | -0.031 | -0.035 | -0.185** | 0.021 | -0.138 |
|  | (0.110) | (0.117) | (0.112) | (0.088) | (0.102) | (0.099) |
| Death in March 2020 or later# COVID-19/respiratory/infectious disease | -0.362*** | -0.050 | -0.041 | -0.292*** | -0.220* | -0.250** |
|  | (0.130) | (0.135) | (0.127) | (0.102) | (0.122) | (0.114) |
| Death in March 2020 or later# Other cause of death | -0.324*** | -0.064 | -0.057 | -0.017 | -0.022 | -0.165 |
|  | (0.123) | (0.132) | (0.120) | (0.102) | (0.121) | (0.111) |
| Gender (Male)  Female | -0.120*** | 0.072 | 0.142*** | 0.080** | -0.026 | -0.003 |
|  | (0.043) | (0.048) | (0.043) | (0.036) | (0.043) | (0.039) |
| Age at death (50-74)  75-84 | -0.051 | -0.011 | 0.264*** | 0.144*** | 0.110** | -0.055 |
|  | (0.053) | (0.057) | (0.055) | (0.044) | (0.050) | (0.048) |
| 85+ | -0.164*** | 0.018 | 0.509*** | 0.333*** | 0.119** | -0.293*** |
|  | (0.056) | (0.062) | (0.056) | (0.047) | (0.054) | (0.050) |
| Self-rated health (poor/fair)  good | -0.040 | 0.047 | -0.111** | -0.217*** | -0.099** | -0.123*** |
|  | (0.050) | (0.058) | (0.052) | (0.044) | (0.050) | (0.046) |
| very good/excellent | -0.024 | 0.126 | -0.141 | -0.308*** | -0.296*** | -0.221*** |
|  | (0.085) | (0.090) | (0.089) | (0.074) | (0.078) | (0.076) |
| Partnership status (has a partner)  No partner | -0.037 | 0.002 | 0.281*** | 0.012 | 0.174*** | -0.026 |
|  | (0.045) | (0.052) | (0.044) | (0.038) | (0.046) | (0.041) |
| Education level (low)  Medium | 0.108* | -0.022 | -0.105* | 0.016 | 0.052 | 0.192*** |
|  | (0.064) | (0.068) | (0.062) | (0.051) | (0.062) | (0.057) |
| High | -0.026 | -0.034 | -0.034 | -0.032 | -0.038 | 0.309*** |
|  | (0.082) | (0.089) | (0.079) | (0.067) | (0.081) | (0.078) |
| Death before March 2020 # Low education | 0.000 | 0.000 | 0.000 | 0.000 | 0.000 | 0.000 |
|  | (0.000) | (0.000) | (0.000) | (0.000) | (0.000) | (0.000) |
| Death before March 2020 # Medium education | 0.000 | 0.000 | 0.000 | 0.000 | 0.000 | 0.000 |
|  | (0.000) | (0.000) | (0.000) | (0.000) | (0.000) | (0.000) |
| Death before March 2020 # High education | 0.000 | 0.000 | 0.000 | 0.000 | 0.000 | 0.000 |
|  | (0.000) | (0.000) | (0.000) | (0.000) | (0.000) | (0.000) |
| Death in March 2020 or later # Low education | 0.000 | 0.000 | 0.000 | 0.000 | 0.000 | 0.000 |
|  | (0.000) | (0.000) | (0.000) | (0.000) | (0.000) | (0.000) |
| Death in March 2020 or later # Medium education | 0.025 | 0.077 | 0.036 | -0.092 | -0.001 | -0.004 |
|  | (0.089) | (0.102) | (0.089) | (0.076) | (0.087) | (0.080) |
| Death in March 2020 or later # High education | 0.128 | 0.145 | -0.025 | 0.094 | 0.117 | -0.053 |
|  | (0.120) | (0.136) | (0.118) | (0.103) | (0.119) | (0.112) |
| Ability to make ends meet (easily or fairly easily)  with (great) difficulty | -0.057 | 0.079 | -0.040 | 0.009 | 0.006 | -0.016 |
|  | (0.058) | (0.063) | (0.057) | (0.047) | (0.058) | (0.052) |
| Death before March 2020 # Make ends meet (fairly) easily | 0.000 | 0.000 | 0.000 | 0.000 | 0.000 | 0.000 |
|  | (0.000) | (0.000) | (0.000) | (0.000) | (0.000) | (0.000) |
| Death before March 2020 # Make ends meet (fairly) easily | 0.000 | 0.000 | 0.000 | 0.000 | 0.000 | 0.000 |
|  | (0.000) | (0.000) | (0.000) | (0.000) | (0.000) | (0.000) |
| Death in March 2020 or later # Make ends meet with (great) difficulty | 0.000 | 0.000 | 0.000 | 0.000 | 0.000 | 0.000 |
|  | (0.000) | (0.000) | (0.000) | (0.000) | (0.000) | (0.000) |
| Death in March 2020 or later # Make ends meet with (great) difficulty | 0.066 | -0.029 | 0.018 | -0.074 | -0.041 | -0.043 |
|  | (0.081) | (0.094) | (0.081) | (0.069) | (0.081) | (0.073) |
| Country (Austria)  Germany | 0.372** | 0.252 | 0.119 | 0.814*** | 0.288* | 0.410*** |
|  | (0.157) | (0.164) | (0.121) | (0.110) | (0.156) | (0.123) |
| Sweden | -0.322*** | 0.647*** | 0.157 | -0.156 | -0.357*** | -0.037 |
|  | (0.119) | (0.146) | (0.109) | (0.105) | (0.124) | (0.104) |
| Netherlands | -0.296* | 0.314* | 0.327** | 0.434*** | -0.262* | 0.628*** |
|  | (0.156) | (0.188) | (0.144) | (0.132) | (0.159) | (0.159) |
| Spain | -0.086 | 0.303* | -0.332*** | 0.464*** | 0.208 | 0.489*** |
|  | (0.124) | (0.156) | (0.118) | (0.103) | (0.141) | (0.111) |
| Italy | -0.070 | 0.171 | -0.486*** | 0.111 | 0.023 | 0.204** |
|  | (0.120) | (0.153) | (0.119) | (0.100) | (0.127) | (0.103) |
| France | 0.166 | 0.945*** | 0.312** | 0.746*** | 0.315* | 0.398*** |
|  | (0.156) | (0.157) | (0.127) | (0.120) | (0.176) | (0.131) |
| Denmark | -0.175 | 0.312* | 0.536*** | 1.027*** | -0.675*** | -0.454*** |
|  | (0.136) | (0.164) | (0.120) | (0.116) | (0.130) | (0.113) |
| Greece | -0.431*** | -1.185*** | -1.437*** | 0.045 | -0.391*** | 0.379*** |
|  | (0.122) | (0.351) | (0.185) | (0.108) | (0.127) | (0.112) |
| Switzerland | -0.456*** | 0.973*** | 0.637*** | 0.755*** | -0.281* | -0.082 |
|  | (0.146) | (0.166) | (0.135) | (0.130) | (0.156) | (0.133) |
| Belgium | -0.116 | 0.634*** | 0.463*** | 0.361*** | 0.271* | 0.391*** |
|  | (0.123) | (0.146) | (0.108) | (0.102) | (0.141) | (0.109) |
| Israel | 0.191 | -0.099 | -0.372** | 0.708*** | -0.233 | 0.048 |
|  | (0.163) | (0.215) | (0.151) | (0.126) | (0.153) | (0.130) |
| Czech Republic | -0.206* | 0.115 | -0.339*** | 0.214** | -0.049 | 0.652*** |
|  | (0.114) | (0.153) | (0.111) | (0.097) | (0.123) | (0.107) |
| Poland | 0.523*** | 0.211 | -0.895*** | 0.078 | -0.014 | 0.280*** |
|  | (0.134) | (0.150) | (0.132) | (0.098) | (0.123) | (0.101) |
| Luxembourg | 0.061 | 0.445 | 0.285 | -0.138 | -0.353 | 1.129*** |
|  | (0.292) | (0.294) | (0.243) | (0.236) | (0.258) | (0.362) |
| Hungary | 0.076 | 0.237 | -0.087 | -0.326*** | -0.100 | 0.702*** |
|  | (0.139) | (0.170) | (0.125) | (0.120) | (0.140) | (0.129) |
| Portugal | -0.496*** | 1.063*** | -0.074 | 0.928*** | -0.382** | -0.049 |
|  | (0.161) | (0.179) | (0.166) | (0.148) | (0.169) | (0.151) |
| Slovenia | -0.478*** | 0.137 | 0.041 | -0.159 | -0.752*** | -0.130 |
|  | (0.113) | (0.154) | (0.108) | (0.102) | (0.116) | (0.100) |
| Estonia | -0.185* | 0.482*** | 0.043 | 0.081 | -0.491*** | -0.093 |
|  | (0.108) | (0.136) | (0.098) | (0.091) | (0.110) | (0.092) |
| Croatia | -0.091 | 0.581*** | -0.298** | -0.031 | -0.030 | 0.491*** |
|  | (0.134) | (0.157) | (0.133) | (0.116) | (0.140) | (0.123) |
| Lithuania | 0.195 | 0.417** | 0.156 | 0.197 | -0.191 | 0.241* |
|  | (0.175) | (0.188) | (0.142) | (0.134) | (0.163) | (0.143) |
| Bulgaria | -0.379** | 0.053 | -1.152*** | -0.768*** | -0.195 | 0.162 |
|  | (0.176) | (0.250) | (0.316) | (0.214) | (0.188) | (0.165) |
| Cyprus | -0.292 | 0.514** | -0.463** | 0.002 | -0.155 | 0.145 |
|  | (0.210) | (0.256) | (0.230) | (0.193) | (0.232) | (0.196) |
| Finland | 0.847* | 0.615** | 0.085 | 0.480** | -0.747*** | 0.139 |
|  | (0.474) | (0.279) | (0.256) | (0.225) | (0.238) | (0.246) |
| Latvia | 0.362* | 0.325 | -0.497** | 0.439*** | -0.374** | -0.247 |
|  | (0.209) | (0.217) | (0.194) | (0.151) | (0.175) | (0.152) |
| Malta | -0.250 | 0.242 | -0.888*** | -0.321 | -0.657*** | -0.531*** |
|  | (0.218) | (0.280) | (0.298) | (0.214) | (0.204) | (0.192) |
| Romania | 0.052 | -0.004 | -0.745*** | 0.004 | -0.221 | 0.517*** |
|  | (0.156) | (0.204) | (0.178) | (0.130) | (0.151) | (0.141) |
| Slovakia | -0.008 | -^1^ | 0.148 | 0.465** | 0.252 | 0.925*** |
|  | (0.268) |  | (0.243) | (0.205) | (0.286) | (0.307) |
| Constant | 1.522*** | -1.280*** | -1.518*** | -0.540*** | 1.214*** | 0.926*** |
|  | (0.117) | (0.141) | (0.112) | (0.096) | (0.119) | (0.101) |
|  |  |  |  |  |  |  |
| Observations | 6,418 | 6,372 | 6,418 | 6,418 | 6,418 | 6,418 |

Slovakia omitted from the probit estimates because no cases of hospice care use were observed in the sample.

**Table C.2. Average Marginal Effects of the interaction terms of probit regression models of the type of care received at the end of life and sociodemographic characteristics of SHARE decedents aged 50+, n = 6,418, with extended time period (2016-2022)**

| **Variables** | **Hospital care** | **Hospice care** | **Nursing home care** | **Home care** | **GP care** | **Specialist physician care** |
| --- | --- | --- | --- | --- | --- | --- |
| Death in March 2020 or later#cancer death | 0.004 | -0.012 | -0.013 | -0.039* | -0.028 | -0.012 |
|  | (0.015) | (0.019) | (0.015) | (0.023) | (0.018) | (0.017) |
| Death in March 2020 or later#cardiovascular death | -0.083*** | -0.010 | 0.011 | -0.101*** | -0.025 | -0.062** |
|  | (0.017) | (0.010) | (0.016) | (0.019) | (0.016) | (0.019) |
| Death in March 2020 or later#COVID-19, respiratory and infectious death | -0.075*** | -0.015 | 0.009 | -0.141*** | -0.077*** | -0.093*** |
|  | (0.020) | (0.015) | (0.022) | (0.027) | (0.020) | (0.025) |
| Death in March 2020 or later#other death | -0.092*** | -0.019 | 0.006 | -0.041 | -0.032 | -0.076** |
|  | (0.025) | (0.016) | (0.027) | (0.027) | (0.021) | (0.027) |
| Death in March 2020 or later#low education | -0.073*** | -0.021** | 0.008 | -0.080*** | -0.041*** | -0.061*** |
|  | (0.014) | (0.010) | (0.014) | (0.017) | (0.013) | (0.016) |
| Death in March 2020 or later#medium education | -0.048*** | -0.008 | 0.016 | 0.099*** | -0.041** | -0.048** |
|  | (0.014) | (0.013) | (0.015) | (0.019) | (0.016) | (0.017) |
| Death in March 2020 or later#high education | -0.033 | 0.003 | -0.002 | -0.032 | 0.011 | -0.058* |
|  | (0.024) | (0.021) | (0.026) | (0.031) | (0.025) | (0.026) |
| Death in March 2020 or later#make ends meet (fairly) easily | -0.063*** | -0.011 | 0.009 | -0.065*** | -0.029** | -0.050** |
|  | (0.013) | (0.011) | (0.014) | (0.016) | (0.013) | (0.015) |
| Death in March 2020 or later#with (great) difficulty | -0.055*** | -0.017 | 0.011 | -0.096*** | -0.045*** | -0.063*** |
|  | (0.013) | (0.011) | (0.013) | (0.017) | (0.013) | (0.015) |

Note: *The table shows average marginal effects and standard errors in parentheses, of the interaction terms of the probit regression models. Each column reports the results of the probit regression model of each outcome variable. The models control for gender, age, health status, partnership status, education level, financial difficulties, and country. Statistical significance: * p < 0.1, ** p < 0.05, *** p < 0.001.*

**Table C.3. Coefficients of probit regression models of the type of care received at the end of life and sociodemographic characteristics of SHARE decedents aged 50+, n = 4,742, excluding observations between March and April 2020**

| VARIABLES | **Hospital care** | **Hospice care** | **Nursing home care** | **Home care** | **GP care** | **Specialist physician care** |
| --- | --- | --- | --- | --- | --- | --- |
|  |  |  |  |  |  |  |
| Time of death (Death before March 2020)  Death in March 2020 or later | -0.049 | -0.030 | 0.011 | 0.008 | -0.163 | 0.004 |
|  | (0.125) | (0.119) | (0.122) | (0.098) | (0.116) | (0.111) |
| Cause of death (Cancer)  Cardiovascular | -0.286*** | -0.643*** | 0.278*** | -0.124* | -0.124 | -0.469*** |
|  | (0.090) | (0.094) | (0.090) | (0.071) | (0.085) | (0.081) |
| COVID19 or respiratory or infectious | -0.065 | -0.505*** | 0.355*** | 0.056 | 0.056 | -0.283*** |
|  | (0.119) | (0.122) | (0.112) | (0.091) | (0.116) | (0.105) |
| Other | -0.372*** | -0.484*** | 0.492*** | -0.106 | -0.106 | -0.607*** |
|  | (0.103) | (0.109) | (0.099) | (0.086) | (0.104) | (0.094) |
| Death before March 2020 # Cancer | 0.000 | 0.000 | 0.000 | 0.000 | 0.000 | 0.000 |
|  | (0.000) | (0.000) | (0.000) | (0.000) | (0.000) | (0.000) |
| Death before March 2020 # Cardiovascular diseases | 0.000 | 0.000 | 0.000 | 0.000 | 0.000 | 0.000 |
|  | (0.000) | (0.000) | (0.000) | (0.000) | (0.000) | (0.000) |
| Death before March 2020 # COVID-19/respiratory/infectious disease | 0.000 | 0.000 | 0.000 | 0.000 | 0.000 | 0.000 |
|  | (0.000) | (0.000) | (0.000) | (0.000) | (0.000) | (0.000) |
| Death before March 2020 # Other cause of death | 0.000 | 0.000 | 0.000 | 0.000 | 0.000 | 0.000 |
|  | (0.000) | (0.000) | (0.000) | (0.000) | (0.000) | (0.000) |
| Death in March 2020 or later# Cancer | 0.000 | 0.000 | 0.000 | 0.000 | 0.000 | 0.000 |
|  | (0.000) | (0.000) | (0.000) | (0.000) | (0.000) | (0.000) |
| Death in March 2020 or later# Cardiovascular diseases | -0.253** | -0.055 | 0.002 | -0.194* | 0.066 | -0.041 |
|  | (0.127) | (0.133) | (0.127) | (0.101) | (0.117) | (0.113) |
| Death in March 2020 or later# COVID-19/respiratory/infectious disease | -0.300** | -0.091 | -0.048 | -0.314*** | -0.182 | -0.230* |
|  | (0.152) | (0.159) | (0.148) | (0.119) | (0.144) | (0.134) |
| Death in March 2020 or later# Other cause of death | -0.306** | -0.078 | 0.111 | -0.080 | 0.031 | -0.111 |
|  | (0.142) | (0.152) | (0.138) | (0.118) | (0.140) | (0.129) |
| Gender (Male)  Female | -0.131*** | 0.074 | 0.147*** | 0.103** | -0.021 | -0.008 |
|  | (0.050) | (0.057) | (0.050) | (0.043) | (0.049) | (0.045) |
| Age at death (50-74)  75-84 | -0.053 | 0.076 | 0.305*** | 0.180*** | 0.105* | -0.029 |
|  | (0.062) | (0.067) | (0.065) | (0.052) | (0.059) | (0.056) |
| 85+ | -0.193*** | 0.029 | 0.589*** | 0.388*** | 0.075 | -0.296*** |
|  | (0.064) | (0.074) | (0.066) | (0.055) | (0.063) | (0.058) |
| Self-rated health (poor/fair)  good | -0.052 | 0.019 | -0.130** | -0.243*** | -0.122** | -0.107** |
|  | (0.057) | (0.068) | (0.060) | (0.051) | (0.058) | (0.053) |
| very good/excellent | 0.033 | 0.074 | -0.124 | -0.266*** | -0.389*** | -0.204** |
|  | (0.099) | (0.108) | (0.103) | (0.086) | (0.089) | (0.088) |
| Partnership status (has a partner)  No partner | -0.016 | 0.027 | 0.291*** | 0.019 | 0.170*** | -0.014 |
|  | (0.052) | (0.061) | (0.051) | (0.045) | (0.053) | (0.047) |
| Education level (low)  Medium | 0.143* | 0.016 | -0.080 | 0.014 | 0.074 | 0.242*** |
|  | (0.082) | (0.086) | (0.078) | (0.065) | (0.079) | (0.072) |
| High | 0.040 | -0.036 | 0.007 | -0.072 | -0.073 | 0.335*** |
|  | (0.107) | (0.117) | (0.102) | (0.088) | (0.103) | (0.099) |
| Death before March 2020 # Low education | 0.000 | 0.000 | 0.000 | 0.000 | 0.000 | 0.000 |
|  | (0.000) | (0.000) | (0.000) | (0.000) | (0.000) | (0.000) |
| Death before March 2020 # Medium education | 0.000 | 0.000 | 0.000 | 0.000 | 0.000 | 0.000 |
|  | (0.000) | (0.000) | (0.000) | (0.000) | (0.000) | (0.000) |
| Death before March 2020 # High education | 0.000 | 0.000 | 0.000 | 0.000 | 0.000 | 0.000 |
|  | (0.000) | (0.000) | (0.000) | (0.000) | (0.000) | (0.000) |
| Death in March 2020 or later # Low education | 0.000 | 0.000 | 0.000 | 0.000 | 0.000 | 0.000 |
|  | (0.000) | (0.000) | (0.000) | (0.000) | (0.000) | (0.000) |
| Death in March 2020 or later # Medium education | -0.011 | 0.043 | 0.039 | -0.087 | -0.053 | -0.083 |
|  | (0.103) | (0.117) | (0.102) | (0.087) | (0.101) | (0.092) |
| Death in March 2020 or later # High education | 0.074 | 0.148 | -0.068 | 0.193 | 0.160 | -0.114 |
|  | (0.140) | (0.158) | (0.136) | (0.119) | (0.138) | (0.129) |
| Ability to make ends meet (easily or fairly easily)  with (great) difficulty | 0.013 | 0.137* | 0.004 | 0.120** | 0.023 | 0.085 |
|  | (0.075) | (0.081) | (0.072) | (0.061) | (0.074) | (0.067) |
| Death before March 2020 # Make ends meet (fairly) easily | 0.000 | 0.000 | 0.000 | 0.000 | 0.000 | 0.000 |
|  | (0.000) | (0.000) | (0.000) | (0.000) | (0.000) | (0.000) |
| Death before March 2020 # Make ends meet (fairly) easily | 0.000 | 0.000 | 0.000 | 0.000 | 0.000 | 0.000 |
|  | (0.000) | (0.000) | (0.000) | (0.000) | (0.000) | (0.000) |
| Death in March 2020 or later # Make ends meet with (great) difficulty | 0.000 | 0.000 | 0.000 | 0.000 | 0.000 | 0.000 |
|  | (0.000) | (0.000) | (0.000) | (0.000) | (0.000) | (0.000) |
| Death in March 2020 or later # Make ends meet with (great) difficulty | 0.010 | -0.096 | 0.004 | -0.161** | -0.031 | -0.159* |
|  | (0.093) | (0.108) | (0.094) | (0.080) | (0.093) | (0.084) |
| Country (Austria)  Germany | 0.478** | 0.243 | 0.039 | 0.779*** | 0.200 | 0.327** |
|  | (0.187) | (0.204) | (0.143) | (0.132) | (0.184) | (0.146) |
| Sweden | -0.176 | 0.644*** | 0.037 | -0.183 | -0.388*** | -0.083 |
|  | (0.137) | (0.178) | (0.127) | (0.125) | (0.145) | (0.122) |
| Netherlands | -0.377** | 0.416* | 0.326* | 0.479*** | -0.171 | 0.463** |
|  | (0.177) | (0.225) | (0.169) | (0.157) | (0.194) | (0.180) |
| Spain | 0.188 | 0.489** | -0.451*** | 0.497*** | 0.237 | 0.569*** |
|  | (0.153) | (0.191) | (0.143) | (0.127) | (0.177) | (0.137) |
| Italy | -0.072 | 0.230 | -0.557*** | 0.141 | -0.015 | 0.154 |
|  | (0.135) | (0.186) | (0.137) | (0.119) | (0.151) | (0.120) |
| France | 0.146 | 0.978*** | 0.312** | 0.795*** | 0.197 | 0.346** |
|  | (0.178) | (0.194) | (0.150) | (0.146) | (0.204) | (0.156) |
| Denmark | -0.250 | 0.336 | 0.418*** | 0.954*** | -0.817*** | -0.554*** |
|  | (0.157) | (0.205) | (0.144) | (0.141) | (0.156) | (0.138) |
| Greece | -0.416*** | -1.002*** | -1.684*** | -0.133 | -0.470*** | 0.363*** |
|  | (0.139) | (0.373) | (0.229) | (0.129) | (0.150) | (0.131) |
| Switzerland | -0.457*** | 1.064*** | 0.585*** | 0.849*** | -0.319* | -0.252 |
|  | (0.170) | (0.202) | (0.161) | (0.157) | (0.186) | (0.158) |
| Belgium | -0.154 | 0.607*** | 0.377*** | 0.323*** | 0.425** | 0.250** |
|  | (0.138) | (0.179) | (0.125) | (0.121) | (0.177) | (0.125) |
| Israel | 0.127 | -0.285 | -0.601*** | 0.776*** | -0.309* | -0.085 |
|  | (0.184) | (0.295) | (0.187) | (0.152) | (0.181) | (0.155) |
| Czech Republic | 0.115 | -0.013 | -0.383*** | 0.321*** | -0.026 | 0.831*** |
|  | (0.137) | (0.196) | (0.129) | (0.116) | (0.148) | (0.133) |
| Poland | 0.559*** | 0.254 | -0.955*** | 0.105 | -0.119 | 0.214* |
|  | (0.146) | (0.177) | (0.145) | (0.113) | (0.139) | (0.114) |
| Luxembourg | 0.092 | 0.794** | 0.017 | -0.291 | -0.604** | 1.280** |
|  | (0.349) | (0.334) | (0.306) | (0.307) | (0.302) | (0.499) |
| Hungary | 0.045 | 0.382* | -0.108 | -0.471*** | -0.226 | 0.575*** |
|  | (0.157) | (0.203) | (0.144) | (0.149) | (0.163) | (0.148) |
| Portugal | -0.493*** | 0.951*** | -0.182 | 0.917*** | -0.602*** | -0.187 |
|  | (0.183) | (0.216) | (0.191) | (0.171) | (0.190) | (0.172) |
| Slovenia | -0.427*** | 0.159 | -0.043 | -0.189 | -0.858*** | -0.187 |
|  | (0.129) | (0.186) | (0.124) | (0.121) | (0.136) | (0.116) |
| Estonia | -0.142 | 0.542*** | -0.074 | 0.012 | -0.494*** | -0.126 |
|  | (0.122) | (0.166) | (0.113) | (0.108) | (0.131) | (0.108) |
| Croatia | -0.056 | 0.647*** | -0.439*** | -0.074 | -0.136 | 0.406*** |
|  | (0.148) | (0.186) | (0.152) | (0.134) | (0.158) | (0.137) |
| Lithuania | 0.388** | 0.435** | 0.038 | 0.167 | -0.317* | 0.248 |
|  | (0.198) | (0.213) | (0.155) | (0.147) | (0.177) | (0.156) |
| Bulgaria | -0.270 | 0.159 | -1.459*** | -0.723*** | -0.280 | 0.202 |
|  | (0.192) | (0.270) | (0.392) | (0.224) | (0.204) | (0.182) |
| Cyprus | -0.234 | 0.545* | -0.480** | -0.014 | -0.266 | 0.115 |
|  | (0.224) | (0.286) | (0.242) | (0.210) | (0.245) | (0.212) |
| Finland | 0.833* | 0.649** | 0.196 | 0.623** | -0.803*** | 0.160 |
|  | (0.494) | (0.317) | (0.275) | (0.254) | (0.266) | (0.279) |
| Latvia | 0.371* | 0.314 | -0.620*** | 0.381** | -0.498*** | -0.304* |
|  | (0.218) | (0.241) | (0.207) | (0.164) | (0.188) | (0.164) |
| Malta | -0.160 | 0.339 | -0.920*** | -0.316 | -0.637*** | -0.583*** |
|  | (0.231) | (0.297) | (0.306) | (0.228) | (0.221) | (0.204) |
| Romania | 0.072 | 0.063 | -0.830*** | 0.004 | -0.293* | 0.425*** |
|  | (0.166) | (0.224) | (0.191) | (0.143) | (0.166) | (0.152) |
| Slovakia | 0.001 | -^1^ | -0.016 | 0.391* | 0.150 | 0.853*** |
|  | (0.278) |  | (0.260) | (0.221) | (0.299) | (0.315) |
| Constant | 1.468*** | -1.386*** | -1.420*** | -0.620*** | 1.317*** | 0.932*** |
|  | (0.141) | (0.176) | (0.136) | (0.119) | (0.146) | (0.124) |
|  |  |  |  |  |  |  |
| Observations | 4,742 | 4,700 | 4,742 | 4,742 | 4,742 | 4,742 |

Slovakia omitted from the probit estimates because no cases of hospice care use were observed in the sample.

**Table C.4. Average Marginal Effects of the interaction terms of probit regression models of the type of care received at the end of life and sociodemographic characteristics of SHARE decedents aged 50+, n = 4,742, excluding observations between March and April 2020**

| **Variables** | **Hospital care** | **Hospice care** | **Nursing home care** | **Home care** | **GP care** | **Specialist physician care** |
| --- | --- | --- | --- | --- | --- | --- |
| Death in March 2020 or later#cancer death | -0.006 | -0.008 | 0.002 | -0.023 | -0.040 | -0.025 |
|  | (0.017) | (0.022) | (0.017) | (0.026) | (0.021) | (0.020) |
| Death in March 2020 or later#cardiovascular death | -0.075*** | -0.012 | 0.004 | -0.091*** | -0.026 | -0.053** |
|  | (0.024) | (0.012) | (0.018) | (0.022) | (0.018) | (0.022) |
| Death in March 2020 or later#COVID-19, respiratory and infectious death | -0.075*** | -0.018 | -0.008 | -0.136*** | -0.080*** | -0.105*** |
|  | (0.023) | (0.018) | (0.025) | (0.032) | (0.023) | (0.028) |
| Death in March 2020 or later#other death | -0.102*** | -0.018 | 0.040 | -0.047 | -0.031 | -0.074 |
|  | (0.029) | (0.019) | (0.030) | (0.032) | (0.024) | (0.032) |
| Death in March 2020 or later#low education | -0.073*** | -0.020† | 0.008 | -0.084*** | -0.043*** | -0.057*** |
|  | (0.016) | (0.012) | (0.016) | (0.019) | (0.014) | (0.018) |
| Death in March 2020 or later#medium education | -0.054*** | -0.012 | 0.015 | -0.095*** | -0.055*** | -0.063*** |
|  | (0.016) | (0.015) | (0.018) | (0.022) | (0.018) | (0.020) |
| Death in March 2020 or later#high education | -0.042 | 0.009 | -0.009 | 0.008 | -0.003 | -0.064*** |
|  | (0.027) | (0.024) | (0.030) | (0.036) | (0.028) | (0.031) |
| Death in March 2020 or later#make ends meet (fairly) easily | -0.062*** | -0.004 | 0.009 | -0.043*** | -0.035*** | -0.039*** |
|  | (0.017) | (0.012) | (0.016) | (0.019) | (0.014) | (0.017) |
| Death in March 2020 or later#with (great) difficulty | -0.062*** | -0.022† | 0.007 | -0.107*** | -0.049*** | -0.082*** |
|  | (0.017) | (0.012) | (0.014) | (0.019) | (0.015) | (0.018) |

Note: *The table shows average marginal effects and standard errors in parentheses, of the interaction terms of the probit regression models. Each column reports the results of the probit regression model of each outcome variable. The models control for gender, age, health status, partnership status, education level, financial difficulties, and country. Statistical significance: * p < 0.1, ** p < 0.05, *** p < 0.001.*

**Table C.5. Coefficients of probit regression models of the type of care received at the end of life and sociodemographic characteristics of SHARE decedents aged 50+, n = 5,029, grouping countries by geographical areas**

| VARIABLES | **Hospital care** | **Hospice care** | **Nursing home care** | **Home care** | **GP care** | **Specialist physician care** |
| --- | --- | --- | --- | --- | --- | --- |
|  |  |  |  |  |  |  |
| Time of death (Death before March 2020)  Death in March 2020 or later | 0.021 | -0.062 | -0.002 | -0.071 | -0.150 | 0.030 |
|  | (0.119) | (0.114) | (0.117) | (0.093) | (0.111) | (0.106) |
| Cause of death (Cancer)  Cardiovascular | -0.239*** | -0.615*** | 0.234*** | -0.210*** | -0.098 | -0.438*** |
|  | (0.086) | (0.088) | (0.085) | (0.068) | (0.081) | (0.078) |
| COVID19 or respiratory or infectious | -0.071 | -0.555*** | 0.245** | -0.014 | 0.073 | -0.217** |
|  | (0.112) | (0.116) | (0.106) | (0.086) | (0.110) | (0.100) |
| Other | -0.383*** | -0.471*** | 0.542*** | -0.159* | -0.129 | -0.598*** |
|  | (0.099) | (0.104) | (0.094) | (0.081) | (0.099) | (0.090) |
| Death before March 2020 # Cancer | 0.000 | 0.000 | 0.000 | 0.000 | 0.000 | 0.000 |
|  | (0.000) | (0.000) | (0.000) | (0.000) | (0.000) | (0.000) |
| Death before March 2020 # Cardiovascular diseases | 0.000 | 0.000 | 0.000 | 0.000 | 0.000 | 0.000 |
|  | (0.000) | (0.000) | (0.000) | (0.000) | (0.000) | (0.000) |
| Death before March 2020 # COVID-19/respiratory/infectious disease | 0.000 | 0.000 | 0.000 | 0.000 | 0.000 | 0.000 |
|  | (0.000) | (0.000) | (0.000) | (0.000) | (0.000) | (0.000) |
| Death before March 2020 # Other cause of death | 0.000 | 0.000 | 0.000 | 0.000 | 0.000 | 0.000 |
|  | (0.000) | (0.000) | (0.000) | (0.000) | (0.000) | (0.000) |
| Death in March 2020 or later# Cancer | 0.000 | 0.000 | 0.000 | 0.000 | 0.000 | 0.000 |
|  | (0.000) | (0.000) | (0.000) | (0.000) | (0.000) | (0.000) |
| Death in March 2020 or later# Cardiovascular diseases | -0.256** | -0.071 | 0.014 | -0.112 | 0.060 | -0.081 |
|  | (0.121) | (0.125) | (0.120) | (0.096) | (0.112) | (0.108) |
| Death in March 2020 or later# COVID-19/respiratory/infectious disease | -0.294** | 0.001 | 0.011 | -0.263** | -0.164 | -0.259** |
|  | (0.144) | (0.149) | (0.139) | (0.112) | (0.137) | (0.128) |
| Death in March 2020 or later# Other cause of death | -0.259* | -0.038 | 0.044 | 0.041 | 0.072 | -0.117 |
|  | (0.135) | (0.143) | (0.131) | (0.111) | (0.133) | (0.123) |
| Gender (Male)  Female | -0.131*** | 0.050 | 0.148*** | 0.067* | -0.047 | -0.017 |
|  | (0.047) | (0.054) | (0.047) | (0.040) | (0.047) | (0.043) |
| Age at death (50-74)  75-84 | -0.066 | 0.056 | 0.288*** | 0.172*** | 0.091 | -0.020 |
|  | (0.060) | (0.064) | (0.062) | (0.049) | (0.056) | (0.053) |
| 85+ | -0.234*** | 0.033 | 0.568*** | 0.374*** | 0.056 | -0.291*** |
|  | (0.061) | (0.069) | (0.062) | (0.052) | (0.060) | (0.055) |
| Self-rated health (poor/fair)  good | -0.114** | 0.020 | -0.126** | -0.248*** | -0.108** | -0.070 |
|  | (0.054) | (0.062) | (0.055) | (0.048) | (0.054) | (0.049) |
| very good/excellent | -0.069 | 0.072 | -0.109 | -0.253*** | -0.340*** | -0.167** |
|  | (0.093) | (0.098) | (0.095) | (0.080) | (0.084) | (0.083) |
| Partnership status (has a partner)  No partner | -0.030 | 0.042 | 0.303*** | 0.001 | 0.155*** | -0.033 |
|  | (0.049) | (0.057) | (0.048) | (0.042) | (0.050) | (0.045) |
| Education level (low)  Medium | 0.179** | -0.007 | 0.002 | -0.016 | -0.017 | 0.162** |
|  | (0.077) | (0.080) | (0.073) | (0.061) | (0.074) | (0.068) |
| High | 0.067 | -0.092 | 0.070 | -0.065 | -0.127 | 0.312*** |
|  | (0.101) | (0.110) | (0.096) | (0.083) | (0.098) | (0.094) |
| Death before March 2020 # Low education | 0.000 | 0.000 | 0.000 | 0.000 | 0.000 | 0.000 |
|  | (0.000) | (0.000) | (0.000) | (0.000) | (0.000) | (0.000) |
| Death before March 2020 # Medium education | 0.000 | 0.000 | 0.000 | 0.000 | 0.000 | 0.000 |
|  | (0.000) | (0.000) | (0.000) | (0.000) | (0.000) | (0.000) |
| Death before March 2020 # High education | 0.000 | 0.000 | 0.000 | 0.000 | 0.000 | 0.000 |
|  | (0.000) | (0.000) | (0.000) | (0.000) | (0.000) | (0.000) |
| Death in March 2020 or later # Low education | 0.000 | 0.000 | 0.000 | 0.000 | 0.000 | 0.000 |
|  | (0.000) | (0.000) | (0.000) | (0.000) | (0.000) | (0.000) |
| Death in March 2020 or later # Medium education | -0.046 | 0.057 | -0.000 | -0.081 | -0.018 | -0.042 |
|  | (0.099) | (0.110) | (0.097) | (0.082) | (0.097) | (0.088) |
| Death in March 2020 or later # High education | 0.025 | 0.182 | -0.044 | 0.124 | 0.157 | -0.084 |
|  | (0.134) | (0.150) | (0.129) | (0.112) | (0.131) | (0.124) |
| Ability to make ends meet (easily or fairly easily)  with (great) difficulty | 0.003 | 0.086 | -0.082 | 0.015 | 0.006 | 0.095 |
|  | (0.069) | (0.075) | (0.067) | (0.056) | (0.069) | (0.062) |
| Death before March 2020 # Make ends meet (fairly) easily | 0.000 | 0.000 | 0.000 | 0.000 | 0.000 | 0.000 |
|  | (0.000) | (0.000) | (0.000) | (0.000) | (0.000) | (0.000) |
| Death before March 2020 # Make ends meet (fairly) easily | 0.000 | 0.000 | 0.000 | 0.000 | 0.000 | 0.000 |
|  | (0.000) | (0.000) | (0.000) | (0.000) | (0.000) | (0.000) |
| Death in March 2020 or later # Make ends meet with (great) difficulty | 0.000 | 0.000 | 0.000 | 0.000 | 0.000 | 0.000 |
|  | (0.000) | (0.000) | (0.000) | (0.000) | (0.000) | (0.000) |
| Death in March 2020 or later # Make ends meet with (great) difficulty | 0.014 | -0.053 | -0.010 | -0.105 | -0.035 | -0.161** |
|  | (0.089) | (0.101) | (0.088) | (0.075) | (0.089) | (0.081) |
| Geographical area (Eastern Europe)  Northern Europe | -0.212*** | 0.351*** | 0.545*** | 0.168*** | -0.386*** | -0.650*** |
|  | (0.077) | (0.087) | (0.074) | (0.063) | (0.072) | (0.067) |
| Southern Europe | -0.367*** | 0.209*** | 0.214*** | 0.056 | -0.279*** | -0.388*** |
|  | (0.059) | (0.070) | (0.062) | (0.049) | (0.057) | (0.054) |
| Western Europe | -0.213*** | 0.396*** | 0.757*** | 0.347*** | 0.170** | -0.262*** |
|  | (0.071) | (0.080) | (0.069) | (0.058) | (0.074) | (0.065) |
| Constant | 1.637*** | -1.198*** | -1.929*** | -0.412*** | 1.243*** | 1.383*** |
|  | (0.106) | (0.109) | (0.109) | (0.084) | (0.100) | (0.095) |
|  |  |  |  |  |  |  |
| Observations | 5,029 | 5,029 | 5,029 | 5,029 | 5,029 | 5,029 |

**Table C.6. Average Marginal Effects of the interaction terms of probit regression models of the type of care received at the end of life and sociodemographic characteristics of SHARE decedents aged 50+, n = 5,029, grouping countries by geographical areas**

| **Variables** | **Hospital care** | **Hospice care** | **Nursing home care** | **Home care** | **GP care** | **Specialist physician care** |
| --- | --- | --- | --- | --- | --- | --- |
| Death in March 2020 or later#cancer death | 0.003 | -0.008 | -0.003 | -0.048* | -0.035* | -0.016 |
|  | (0.016) | (0.022) | (0.017) | (0.026) | (0.020) | (0.019) |
| Death in March 2020 or later#cardiovascular death | -0.062*** | -0.014 | 0.000 | -0.087*** | -0.023 | -0.054** |
|  | (0.018) | (0.011) | (0.018) | (0.022) | (0.017) | (0.021) |
| Death in March 2020 or later#COVID-19, respiratory and infectious death | -0.063*** | -0.007 | 0.000 | -0.146*** | -0.073*** | -0.104*** |
|  | (0.023) | (0.017) | (0.025) | (0.032) | (0.023) | (0.028) |
| Death in March 2020 or later#other death | -0.074*** | -0.013 | 0.011 | -0.029 | -0.018 | -0.064** |
|  | (0.028) | (0.019) | (0.030) | (0.032) | (0.024) | (0.031) |
| Death in March 2020 or later#low education | -0.052*** | -0.019* | 0.003 | -0.081*** | -0.040*** | -0.059*** |
|  | (0.015) | (0.012) | (0.015) | (0.019) | (0.014) | (0.018) |
| Death in March 2020 or later#medium education | -0.045*** | -0.009 | 0.003 | -0.098*** | -0.043** | -0.052** |
|  | (0.016) | (0.015) | (0.017) | (0.022) | (0.017) | (0.019) |
| Death in March 2020 or later#high education | -0.035 | 0.015 | -0.008 | -0.019 | 0.000 | -0.056* |
|  | (0.027) | (0.023) | (0.029) | (0.036) | (0.028) | (0.030) |
| Death in March 2020 or later#make ends meet (fairly) easily | -0.048*** | -0.005 | 0.002 | -0.056*** | -0.028* | -0.033* |
|  | (0.014) | (0.012) | (0.016) | (0.019) | (0.014) | (0.017) |
| Death in March 2020 or later#with (great) difficulty | -0.047*** | -0.017 | 0.000 | -0.101*** | -0.043*** | -0.080*** |
|  | (0.015) | (0.012) | (0.014) | (0.019) | (0.015) | (0.017) |

Note: *The table shows average marginal effects and standard errors in parentheses, of the interaction terms of the probit regression models. Each column reports the results of the probit regression model of each outcome variable. The models control for gender, age, health status, partnership status, education level, financial difficulties, and country. Statistical significance: * p < 0.1, ** p < 0.05, *** p < 0.001.*

1. Slovakia omitted from the probit estimates because no cases of hospice care use were observed in the sample.
